# Automated cold vapour flow-injection analysis of mercury at high concentrations

**DOI:** 10.1155/S1463924691000421

**Published:** 1991

**Authors:** W. T. Corns, L. C. Ebdon, S. J. Hill, P. B. Stockwell

**Affiliations:** 1 Plymouth Analytical Chemistry Research Unit Department of Environmental Sciences Polytechnic Southwest Drake Circus, Devon Plymouth PL4 8AA UK; 2 PS Analytical Ltd Arthur House B4 Chaucer Business Park Watery Lane, Kemsing, Kent Sevenoaks TN15 6QY UK

## Abstract

Continuous-flow cold vapour- atomic fluorescence spectrometry is shown to be an extremely sensitive technique for the determination of mercury with detection limits typically below 0.01 μg l^-1^. Linear calibration ranges were found to be at least four orders of magnitude (i.e. up to 0.1 mg l^-1^). Samples with concentrations exceeding the linear range are susceptible to self-absorption, and may, in severe cases, cause carry-over problems between samples. The flow-injection approach has been utilized to extend the upper limit of the linear calibration range allowing determinations up to 10 mg l^-1^ of mercury. A range of certified reference materials and zinc battery anodes have been successfully analysed with a minimal number of sample dilutions.


Journal of Automatic Chemistry, Vol. 13, No. 6 (November–December 1991), pp. 267–271

### Automated cold vapour flow-injection analysis of mercury at high concentrations

**W. T. Corns, L. C. Ebdon, S. J. Hill**

*Plymouth Analytical Chemistry Research Unit, Department of Environmental Sciences, Polytechnic Southwest, Drake Circus, Plymouth, Devon PL4 8AA, UK*

**and P. B. Stockwell**

*PS Analytical Ltd, Arthur House, B4 Chaucer Business Park, Watery Lane, Kemsing, Sevenoaks, Kent TN15 6QY, UK*

*Continuous-flow cold vapour – atomic fluorescence spectrometry is shown to be an extremely sensitive technique for the determination of mercury with detection limits typically below 0·01 $\mu g\ l^{-1}$. Linear calibration ranges were found to be at least four orders of magnitude (i.e. up to 0·1 $mg\ l^{-1}$). Samples with concentrations exceeding the linear range are susceptible to self-absorption, and may, in severe cases, cause carry-over problems between samples. The flow-injection approach has been utilized to extend the upper limit of the linear calibration range allowing determinations up to 10 $mg\ l^{-1}$ of mercury. A range of certified reference materials and zinc battery anodes have been successfully analysed with a minimal number of sample dilutions.*

### Introduction

The toxicological effect of mercury compounds on both plant and animal life has long been recognized, but it was not until the disaster at Minamata Bay in 1953 that the subject received world-wide attention [1]. Mercury occurs naturally in the environment in the form of mineral deposits and also anthropogenically from industrial and agricultural wastes. Because of its high toxicity, there has been extensive research and development into techniques which can be used to determine mercury in a variety of samples.

Colorimetric and spectrophotometric methods were initially adopted [2], but these had poor sensitivity and entailed long and delicate manipulations which often caused contamination and loss of analyte. Today, the most commonly used method for measuring mercury is cold vapour – atomic absorption spectrometry (CV-AAS). This technique was first described by Poluektov and Vitkun as early as 1963 [3] and later popularized by Hatch and Ott [4]. Since that time, numerous modifications have been reported [5,6] and several commercial systems are now available.

Although CV-AAS has now become a widely used technique, there are several disadvantages associated with the use of AAS detection, such as limited linear calibration range, spectral interferences resulting from non-specific background absorption of volatile organics [7], and difficulties with measurements at lower levels.

Mercury is a good element for determination by fluorescence, since it is atomic at room temperature and also absorbs and fluoresces at the same wavelength (i.e. resonance fluorescence). Intense mercury sources are readily available and atomization cells are simple when the technique is coupled to vapour generation. Thompson and Godden [8] proposed the use of fluorescence for the analysis of mercury in 1975. Like most of the early workers they modified a conventional atomic absorption

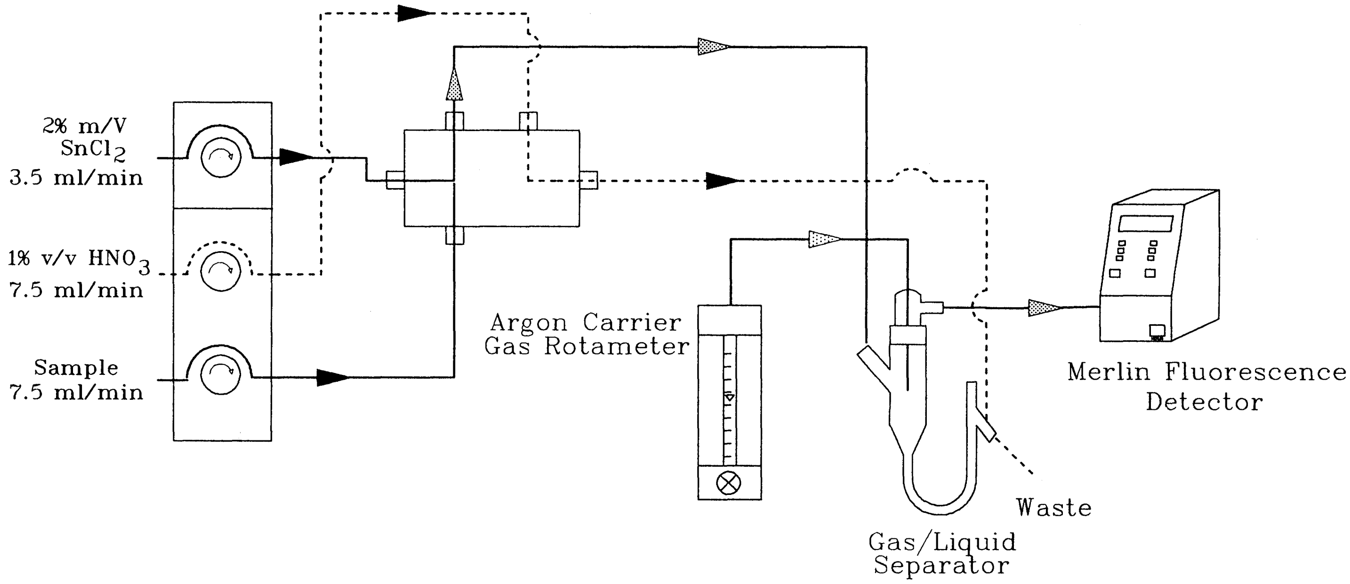

*Figure 1. Schematic diagram of the continuous-flow vapour generation system, shown in the sampling position. (Dotted line represents the flow of the blank solution.)*

0142–0453/91 $3.00 © 1991 Taylor & Francis Ltd.

spectrometer to obtain a dispersive system. More recently, Godden and Stockwell [9] described the development of a non-dispersive fluorescence spectrometer, which was specifically designed for mercury analysis; detection limits typically below 0·02 μg $l^{-1}$ were readily obtainable with this instrument. The main advantages of this system are that no AA spectrometer is required and it is inexpensive, and simple in both construction and operation. Although this sensitivity has many advantages in terms of trace analysis, problems may arise when samples have high mercury content and difficult matrix composition, such as those from the chlor-alkali industry. In this industry vast quantities of metallic mercury is used as a flowing cathode in the electrolysis of brine, producing chlorine and caustic soda. Using a continuous flow (CV-AFS) system, samples containing high levels of mercury will cause carry-over problems and will be susceptible to self-absorption. This problem can be minimized by utilizing the flow-injection approach which was first adopted in 1975 [10]. This essentially involves the introduction of small sample volumes, typically 50–200 μl, into a continuously-flowing carrier stream. The advantages of using flow-injection are well documented with several reviews [11,12] and books [13,14].

This article addresses the problems associated with the analysis of samples containing high levels of mercury. Both the continuous-flow and flow-injection approaches to cold vapour – atomic fluorescence have been investigated with the view of minimizing long carry-over times between samples, self-absorption and matrix interferences. The effect of sample volume on the linear calibration range and sensitivity has been studied. To assess the validity of the flow-injection technique, certified reference materials have been analysed along with commercially available battery anodes.

### Experimental

### *Reagents*

All reagents used were of analytical reagent grade (from Aldrich Chemical Company Ltd, UK), unless otherwise stated. Purified water was used throughout and was obtained from a distillation unit (Jencons Scientific Ltd, Bedfordshire, UK). A 2% m/v stannous chloride solution was used as the reductant and traces of mercury were removed from this solution by purging with argon gas for approximately 20 min. Standard solutions were prepared by appropriate dilution of stock 1000 mg $l^{-1}$ mercury (II) chloride using 1% nitric acid. All glassware was soaked in 10% nitric acid for at least 24 h prior to use, and was then rinsed five times with distilled water.

### *Instrumentation*

Automated continuous-flow and flow-injection vapour generation systems (the PSA 10.003, 40.630, from PS Analytical Ltd, Sevenoaks, Kent, UK) were used to generate gaseous mercury. A schematic diagram of the continuous-flow system is shown in figure 1. Essentially, it consists of a constant-speed peristaltic pump which delivers stannous chloride reductant and acidified sample/blank solutions to a mixing piece. Incorporated into the system is an electrically-controlled switching valve which alternates between sample and blank solutions. The reaction begins at the mixing piece where the two flows meet. This mixture then falls into a gas/liquid separator, where the gaseous products are purged out of solution to the detector. Meanwhile, the liquid products continuously flow to waste [15]. Figure 1 shows the system in the sampling mode – the dotted line represents the flow of the blank solution. A schematic diagram of the

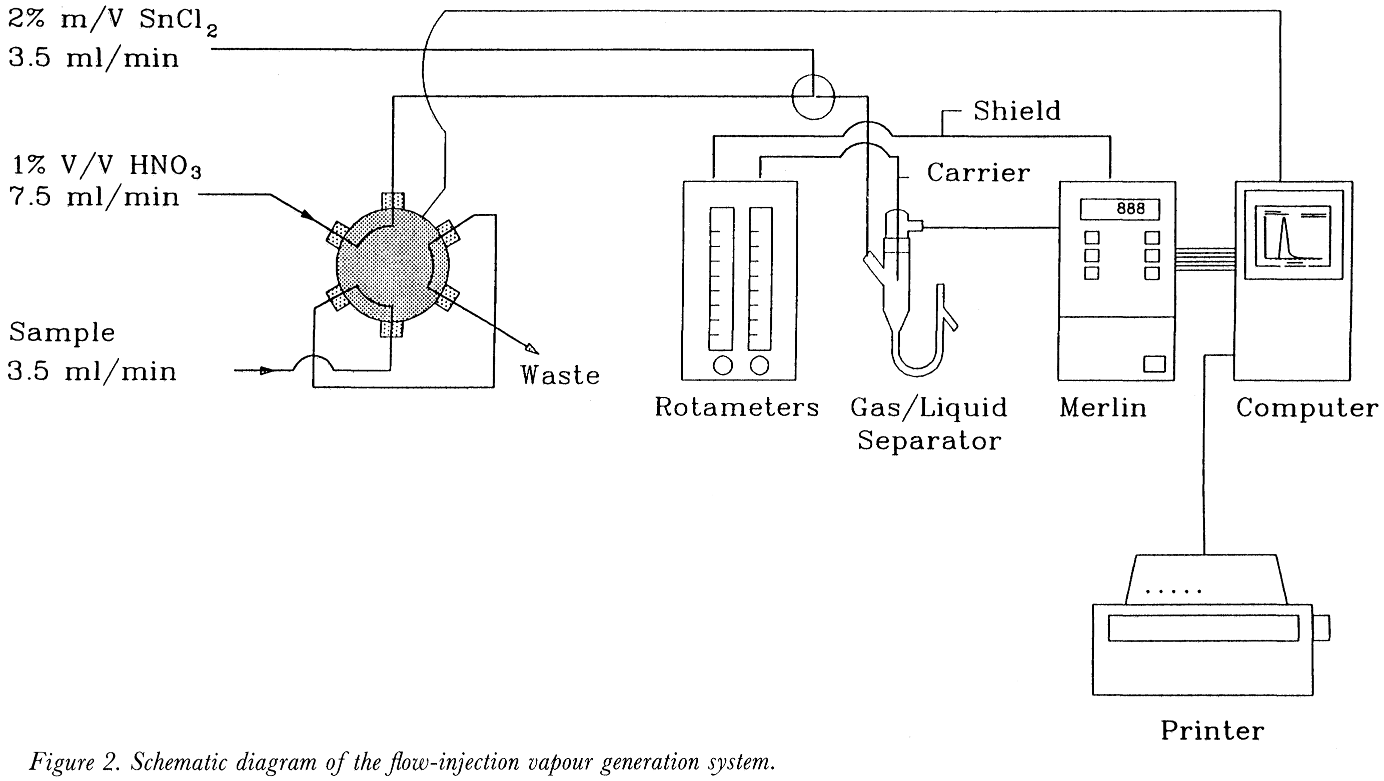

*Figure 2. Schematic diagram of the flow-injection vapour generation system.*

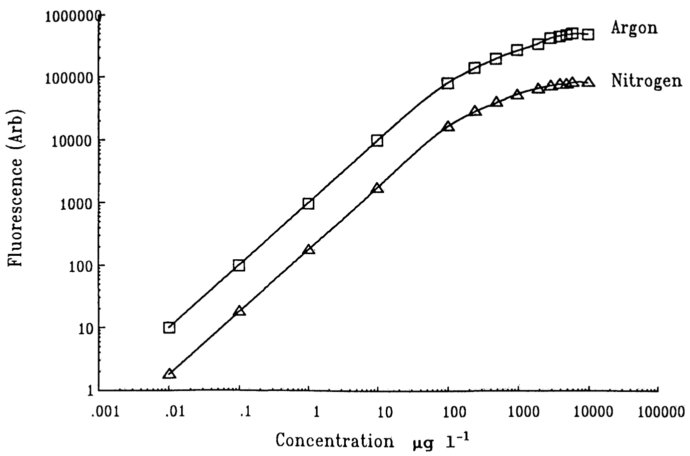

*Figure 3. Analytical response curves for continuous-flow – atomic fluorescence spectrometry using both argon and nitrogen carrier gases.*

flow-injection manifold is shown in figure 2. This is almost identical to the continuous-flow approach, except that an electronically activated six-port valve is utilized to introduce sample solutions into the carrier stream. The sample loop may be filled manually via a syringe method, or by continuously pumping the sample through the loop. Once activated, the valve switches pneumatically using a compressed gas supply.

The generated mercury gas is then detected using a non-dispersive atomic fluorescence spectrometer (PSA 10.023 Merlin). This consists of a high-intensity vapour discharge lamp, a series of lenses and collimating devices to focus and collect light with minimization of scatter, a 254 nm interference filter to achieve wavelength isolation and a conventional photomultiplier tube. This instrument is described in more detail elsewhere [9]. The system is fully

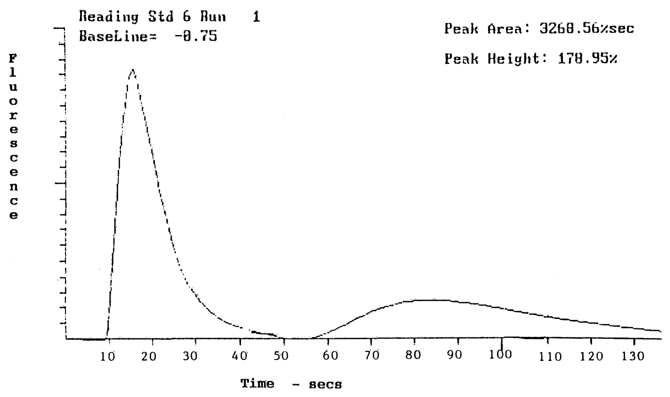

*Figure 5. Typical peak shape using the continuous-flow approach for a 2000 µg $l^{-1}$ solution of mercury illustrating the process of self-absorption.*

automated and controlled using the TouchStone Software (SpinOff Technical Systems, Benfleet, Essex, UK) and was developed in association with PS Analytical Ltd. The different units are connected to an IBM AT compatible computer through a DIO card.

### Results and discussion

*Continuous-flow cold vapour – atomic fluorescence spectrometry*

Fluorescence techniques are extremely sensitive and possess a wide linear calibration range. Figure 3 shows analytical response curves for the continuous-flow approach. Typical limits of detection for this system are below 10 ng $l^{-1}$, with linearity to 100 ng $ml^{-1}$. The linear calibration range therefore stretches over four orders of magnitude, which is obviously beneficial in view of the wide range of mercury concentrations found in the environment. Samples with concentrations exceeding the linearity are susceptible to self-absorption. This process is best explained using a standardized fluorescence cell, like that shown in figure 4.

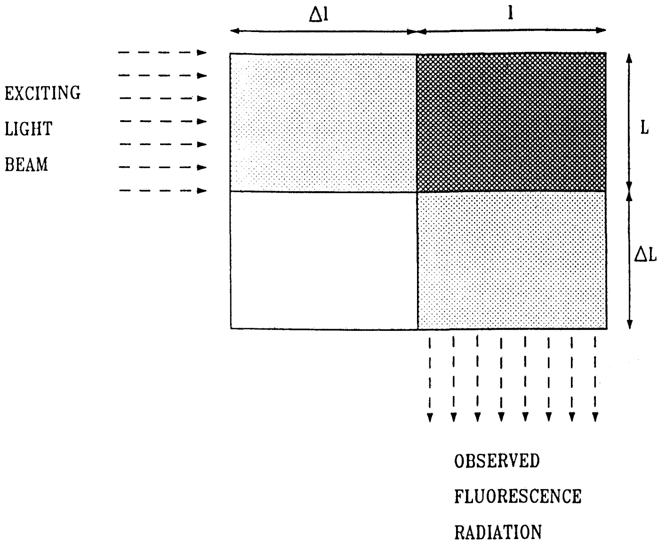

*Figure 4. Standardized atom cell for atomic fluorescence.*

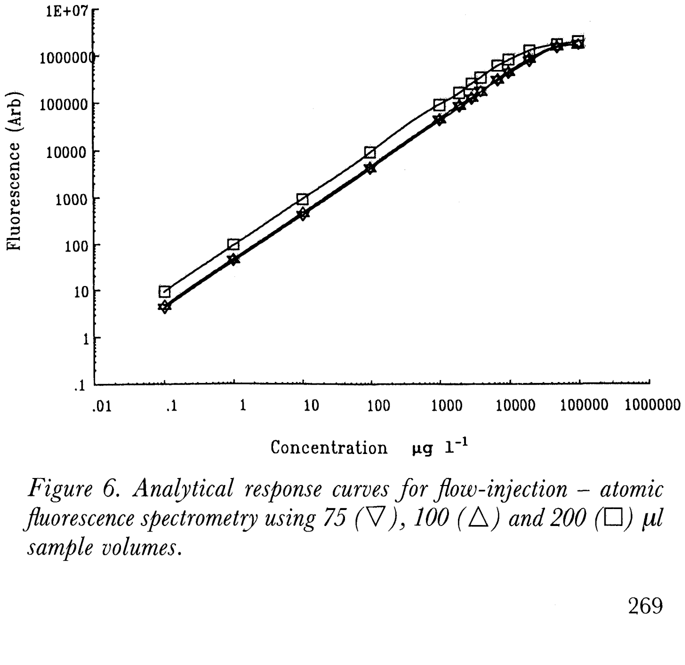

*Figure 6. Analytical response curves for flow-injection – atomic fluorescence spectrometry using 75 (▽), 100 (△) and 200 (□) µl sample volumes.*

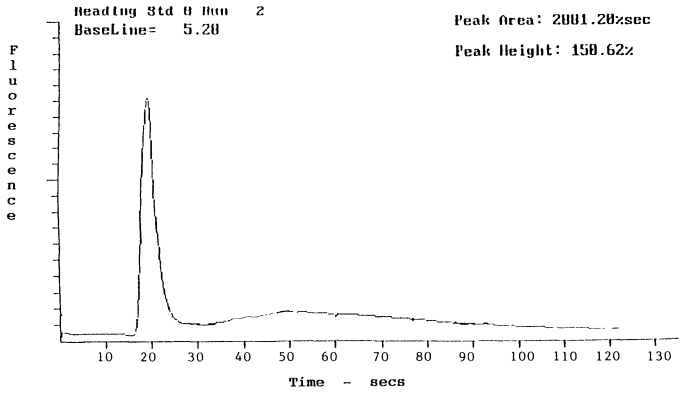

*Figure 7. Typical peak shape for the flow-injection approach for a 100 000 μg $l^{-1}$ solution of mercury, illustrating the process of self-absorption.*

This theoretical model assumes that the light beams are parallel, and that there is uniform atomic concentration and temperature. At high concentrations, incident radiation passing through $\Delta l$ may be lost by absorption before excitation can occur. Useful fluorescence may also be lost by reabsorption in the region $\Delta L$. In an ideal situation, these regions would be infinitely small, thereby minimizing self-absorption. Figure 5 shows a typical profile obtained using the continuous-flow approach for a 2000 μg $l^{-1}$ mercury solution, and the self-absorption process is clearly evident. As the concentration increases, there is a rapid rise in signal until the concentration has reached a level where self-absorption occurs. At this point the signal begins to fall, in severe cases to zero. When the sample is removed and the concentration begins to decline and the signal begins to rise once more. Carry-over times between samples can be up to 5 min, depending on the concentration of mercury present.

The atomic fluorescence signal magnitude can be reduced with the use of alternative carrier gases, such as nitrogen or air. These gases have been found to reduce the fluorescence signal by eight and 30 times respectively, due to quenching (quenching is a deactivation process due to molecular collisions with foreign species):

$$Hg^* + A \rightarrow Hg + A^*.$$

Although a reduction in signal is clearly observed, the quenching process has no relation to linearity because the self-absorption process is dependent on the atomic concentration and the atom cell dimensions. The reduction in signal from quenching therefore has no practical use in this application. The analytical response curve for nitrogen is also shown in figure 3.

*Table 1. Effect of loop size on linear calibration range.*

| Sample volume (μl) | Upper limit calibration range (mg $l^{-1}$) | Slope |
|---|---|---|
| 75 | 10·5 | 3·7 |
| 100 | 10 | 4·4 |
| 200 | 7 | 8·4 |

*Flow-injection approach*

Flow-injection analysis utilizes small sample volumes typically between 50 and 200 μl. Although not as sensitive as the continuous-flow approach, it is less susceptible to self-absorption and matrix interferences. This allows the upper limit of the calibration range to be increased. Figure 6 shows three analytical response curves corresponding to 75, 100 and 200 μl loop sizes. As expected, the smaller volumes gave higher upper limit calibration ranges, with slightly less sensitivity. An estimation of the sensitivity is again obtained from the slope of the curve, at the point where deviation from linearity occurs. Table 1 summarizes the effect of sample volume on linearity.

Samples containing levels of mercury exceeding the linear range are still susceptible to self-absorption; a typical profile is shown in figure 7. The profile corresponds to a 100 000 μg $l^{-1}$ solution of mercury and the self-absorption is clearly observed. However, this is not as severe as that for continuous flow, and the carry-over times between samples with high levels is negligible. This allows the analysis of total samples to proceed with minimal delay.

To assess the validity of the flow-injection CV-AFS technique, a range of certified reference materials and zinc battery anodes have been analysed for mercury. These results are shown in table 2.

Table 2 shows that accurate, precise quantitative measurements can be made using the flow-injection CV-AFS approach. The advantage of this system is that minimal sample dilution is required, which considerably reduces the sample preparation time and errors involved in large serial dilutions. One further advantage is that matrix interference is reduced because the analyte is separated from the matrix by generation of the gas and because small volumes are utilized.

The system described has also been used for the direct determination of mercury in concentrated sulphuric acid. The potential difficulties with this analysis are related to the vigorous exothermic reaction between water and sulphuric acid. The flow-injection approach, however, overcomes these difficulties; in addition, there are no problems related to heat dissipation. A typical response curve within the range of the system is shown in figure 8.

### Conclusions

Continuous-flow cold vapour – atomic fluorescence spectrometry has been shown to be an extremely sensitive technique for the determination of mercury. Atomic fluorescence by its very nature is a linear technique with typical calibration ranges spanning over four orders of magnitude. Samples with concentrations exceeding the linear calibration range are susceptible to self-absorption and these levels can cause large increases in carry-over time between samples. The obvious solution to this is to perform dilutions; however, this increases the sample preparation time and may also give rise to experimental error. The flow-injection approach has been utilized here to increase the linearity of the technique and to overcome

*Table 2. Determination of mercury in certified reference materials and battery anodes.*

| Certified reference material | Expected/certified concentration | Concentration found | Weight dilution |
|---|---|---|---|
| NIST SRM 1641b (Mercury in water) | 1·52 ± 0·04 (μg $ml^{-1}$) | 1·41 ± 0·04 (μg $ml^{-1}$) | 0 |
| NBS SRM 3133 (Spectrometric solution) | 10·00 ± 0·01 (μg $ml^{-1}$) | 9·89 ± 0·20 (μg $ml^{-1}$) | 2500 |
| ZINC ANODE A | 1000 (μg $g^{-1}$) | 1060 ± 30 (μg $g^{-1}$) | 200 |
| ZINC ANODE B | 0 | 4·11 ± 0·29 (μg $g^{-1}$) | 200 |
| ZINC ANODE C | 1200 (μg $g^{-1}$) | 1150 ± 43 (μg $g^{-1}$) | 200 |

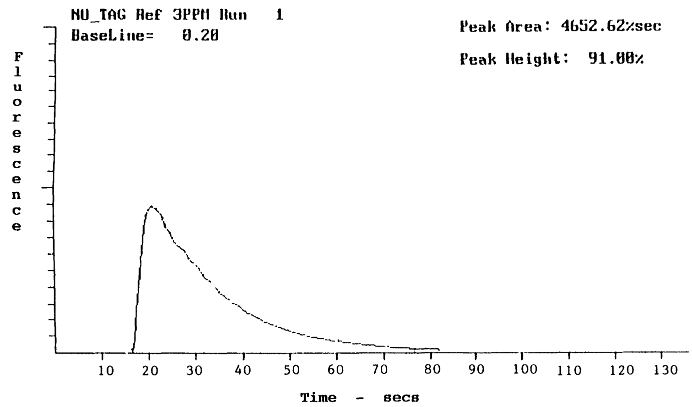

*Figure 8. Typical peak shape using the flow-injection approach for a 3000 μg $l^{-1}$ solution of mercury.*

matrix interference. To assess the potential for the analysis of realistic samples, two certified water reference materials and zinc battery anodes have been analysed.

### Acknowledgements

The authors wish to thank the SERC and PS Analytical for the provision of a SERC-CASE award to one of us (Warren Corns); and D. McGurk, at Duracell, for kindly providing the batttery anode samples.

### References

1. Smith, W. E. and Smith, A. M. (Eds), *Minamata* (Holt, Rinehardt and Winston, New York, 1975).

2. Lindstedt, G., *Analyst*, **95** (1970), 264.

3. Poluektov, N. S. and Vitkun, R. A., *Zh. Anal. Khim.*, **18** (1963), 33.

4. Hatch, W. R. and Ott, W. L., *Analytical Chemistry*, **40**, (1968), 2085.

5. Hawley, J. E. and Ingle, J. D. Jn., *Analytical Chemistry*, **47** (1975), 719.

6. Rooney, R. C., *Analyst*, **101** (1976), 678.

7. Corcoran, F. L., *American Laboratory*, (March 1974).

8. Thompson, K. C. and Godden, R. C., *Analyst*, **100** (1975), 544.

9. Godden, R. G. and Stockwell, P. B., *Journal of Analytical Atomic Spectrometry*, **4** (1989), 301.

10. Růžička, J. and Hansen, E. H., *Analytica Chimica Acta*, **78**, (1975), 31.

11. Stockwell, P. B., *Journal of Automatic Chemistry*, **12** (1990), 95.

12. Luque de Castro, M. D. and Valcárcel, M., *Analyst*, **109** (1984), 413.

13. Růžička, J. and Hansen, E. H., *Flow Injection Analysis*, 2nd edn (John Wiley and Sons, New York, 1988).

14. Valcárcel, M. and Luque de Castro, M. D., *Flow Injection Analysis: Principles and Applications* (Horwood, Chichester, 1987).

15. Ward, R. W. and Stockwell, P. B., *Journal of Automatic Chemistry*, **5** (1983), 193.

